# Functional diversity of voltage‐sensing phosphatases in two urodele amphibians

**DOI:** 10.14814/phy2.12061

**Published:** 2014-07-17

**Authors:** Joshua Mutua, Yuka Jinno, Souhei Sakata, Yoshifumi Okochi, Shuichi Ueno, Hidekazu Tsutsui, Takafumi Kawai, Yasuhiro Iwao, Yasushi Okamura

**Affiliations:** 1 Graduate School of Frontier Biosciences, Osaka University, Suita, Osaka, Japan; 2 Laboratory of Integrative Physiology, Department of Physiology, Graduate School of Medicine, Osaka University, Suita, Osaka, Japan; 3 Laboratory of Molecular Developmental Biology, Department of Applied Molecular Biosciences, Graduate School of Medicine, Yamaguchi University, Yamaguchi, Japan

**Keywords:** C2 domain, phosphoinositides, testis, voltage‐sensing phosphatase

## Abstract

Voltage‐sensing phosphatases (VSPs) share the molecular architecture of the voltage sensor domain (VSD) with voltage‐gated ion channels and the phosphoinositide phosphatase region with the phosphatase and tensin homolog (PTEN), respectively. VSPs enzymatic activities are regulated by the motions of VSD upon depolarization. The physiological role of these proteins has remained elusive, and insights may be gained by investigating biological variations in different animal species. Urodele amphibians are vertebrates with potent activities of regeneration and also show diverse mechanisms of polyspermy prevention. We cloned cDNAs of VSPs from the testes of two urodeles; *Hynobius nebulosus* and *Cynops pyrrhogaster*, and compared their expression and voltage‐dependent activation. Their molecular architecture is highly conserved in both *Hynobius* VSP (Hn‐VSP) and *Cynops* VSP (Cp‐VSP), including the positively‐charged arginine residues in the S4 segment of the VSD and the enzymatic active site for substrate binding, yet the C‐terminal C2 domain of Hn‐VSP is significantly shorter than that of Cp‐VSP and other VSP orthologs. RT‐PCR analysis showed that gene expression pattern was distinct between two VSPs. The voltage sensor motions and voltage‐dependent phosphatase activities were investigated electrophysiologically by expression in *Xenopus* oocytes. Both VSPs showed “sensing” currents, indicating that their voltage sensor domains are functional. The phosphatase activity of Cp‐VSP was found to be voltage dependent, as shown by its ability to regulate the conductance of coexpressed GIRK2 channels, but Hn‐VSP lacked such phosphatase activity due to the truncation of its C2 domain.

## Abstract (precis)

To gain insights into physiological roles of VSP, VSP orthologs were identified from two urodeles, newt and salamander. Cp‐VSP has voltage‐dependent phosphatase activity. Hn‐VSP lacks phosphatase activity due to its truncation in the C2 domain of the cytoplasmic region.

## Introduction

Voltage‐sensing phosphatases (VSPs) consist of the two regions: the voltage sensor domain and the cytoplasmic region that exhibits activities of phosphoinositide phosphatase. VSPs control their enzymatic activity toward phosphoinositides in a voltage‐dependent manner (Murata et al. 2005; Okamura et al. 2009). Originally identified during a genomic survey of the ascidian *Ciona intestinalis*, Ci‐VSP was found to be localized to the sperm flagellum, and later found to be expressed in intestine, stomach, and blood of juveniles of the same species (Ogasawara et al. 2011).

VSPs share a similar molecular architecture of the transmembrane voltage sensor domain (VSD) with Hv1/VSOP and voltage‐gated ion channels (VGICs). The VSD consists of four transmembrane segments, the fourth of which (S4) contains basic amino acid residues critical for the voltage sensitivity. In VGICs, conformational changes of the VSD in response to changes in the membrane potential lead to the gating of the ion‐conducting pore, whereas in VSPs depolarization of membrane potential leads to the dephosphorylation of the phosphoinositides that are localized in the inner leaflet of the lipid bilayer of the plasma membrane. This regulation of phosphatase activity over a wide range of voltage is achieved through a tight coupling of the VSD to the phosphatase domain via the linker region between the two domains (Murata et al. 2005; Villalba‐Galea et al. 2009; Sakata et al. 2011; Sakata and Okamura 2014).

Although the phosphatase region of VSPs resembles that of the phosphatase and tensin homolog (PTEN), their substrate specificity differs from that of PTEN. Through in vitro phosphatase assays, VSPs have been shown to dephosphorylate phosphatidylinositol 3,4,5‐trisphosphate (PtdIns(3,4,5)P_3_) and phosphatidylinositol 4,5‐bisphosphate (PtdIns(4,5)P_2_) at the D5 position of the inositol ring (Murata et al. 2005; Iwasaki et al. 2008; Okamura and Dixon 2011). This differs from PTEN, which dephosphorylates PtdIns(3,4,5)P_3_ by acting on the phosphate at the D3 position (Maehama et al. 2001). In contrast with detailed information of biophysical and enzymatic properties of VSPs, information on their physiological role has remained elusive.

Orthologous cDNAs encoding VSPs have been identified from zebrafish, *Danio rerio* (Hossain et al. 2008); chick, *Gallus gallus* (Kurokawa et al. 2012); and frogs, *Xenopus laevis* and *tropicalis* (Ratzan et al. 2011). Mammalian orthologs (TPTE and TPTE2/TPIP) have been shown to be expressed in spermatocytes (Guipponi et al. 2001; Walker et al. 2001; Tapparel et al. 2003) and in tumors (Pleasance et al. 2010).

Recently, it has been demonstrated that chick VSP (Gg‐VSP) is able to regulate cell shape when ectopically expressed in a fibroblast cell line and that this is mediated by voltage‐sensitive regulation of the level of PtdIns(3,4)P_2_ (Yamaguchi et al. 2014).

In this study, cDNAs encoding VSPs were cloned from the testes of newt and salamander and their molecular properties were compared in *Xenopus* oocytes as the heterologous expression system. Comparison of the amino acid sequence of the newt VSP (named as Cp‐VSP after the species name *Cynops pyrrhogaster*) and salamander VSP (named as Hn‐VSP after *Hynobius nebulosus*) with those of other VSP orthologs showed that their molecular architecture is highly conserved. In Hn‐VSP, approximately two thirds of the C2 domain was missing from the cytoplasmic region. The phosphatase activity of Cp‐VSP was found to be voltage dependent, as shown by its ability to regulate the ion conductance of coexpressed GIRK2 channels. However, Hn‐VSP lacked such phosphatase activity, which was probably due to its truncation of the C2 domain.

## Materials and Methods

### cDNA cloning of VSP orthologs from *C. pyrrhogaster* and *H. nebulosus*

Total RNA was isolated from the testis of a newt (*C. pyrrhogaster*) with TRIzol^®^ reagent (Invitrogen, Carlsbad, CA) according to the manufacturer's protocol. Using Superscript^™^ III First Strand cDNA synthesis kit (Invitrogen), cDNA was synthesized from 1 *μ*g of the total RNA of testis with oligo(dT)_20_. The primers used in this study are listed in Table 1. A 600‐bp fragment of Cp‐VSP was cloned by RT‐PCR using the degenerate primers #1 and 2 followed by nested PCR with degenerate primers #3 and 4. The target fragment was cloned into pCR4TOPO vector (Invitrogen). The nucleotide sequence information obtained from this fragment was used to amplify the flanking 5′ and 3′ ends of the cDNA with a GeneRacer^™^ kit (Invitrogen), using the RLM‐RACE Version L protocol. The 3′ side of the cDNA fragment was cloned by the method of rapid amplification of cDNA ends (RACE) with reverse primer GeneRacer^™^ 3′ and forward gene‐specific primer #5. Nested PCR was performed with reverse GeneRacer^™^ 3′ nested primer and forward gene‐specific nested primer #6. A fragment of 1.3 kb was obtained and cloned into the pCR4TOPO vector. Since the 3′ end lacked the poly (A)+ tail, another primer set was employed: forward gene‐specific primer #7 and reverse GeneRacer^™^ 3′ primer. Nested PCR was performed with forward nested gene‐specific primer #8 and reverse GeneRacer^™^ 3′ nested primer. The resulting 1.4‐kb fragment contained the poly (A)+ tail. By the 5′ RACE method, 5′ side cDNA fragment was obtained. GeneRacer^™^ 5′ RNA oligo was ligated to the 5′ end of the mRNA so as to provide a known priming site. First strand was synthesized with gene‐specific primer #9. The 5′ end region was amplified from the RACE cDNA pool with the primer set GeneRacer^™^ 5′ and reverse gene‐specific primer #10. Since the resulting DNA fragment lacked the 5′ untranslated region (UTR) sequence, nested PCR was done with nested primer GeneRacer^™^ 5′ and nested reverse gene‐specific primer #11. A cDNA fragment of 424 bp that contained the 5′ UTR and the ligated RNA Oligo sequence was obtained. Sequence information obtained from both the 5′ and 3′ ends were used to amplify the complete open reading frame (ORF). An ORF of 1536 bp was amplified with the primers #12 and 13 followed by a second PCR with primers #14 and 15.

**Table 1.** List of primers used in the cloning and molecular constructs

#	Forward sequence (5′–3′)	Reverse sequence (5′–3′)	Corresponding amino acids
1	CCNTTTGTGATGTCCTTTGGNTT		PFVMSFGF (D)
2		NGTGTTGAACCAGAANTA	YFWFNT (D)
3	GANAACAAAAGACGNTANCAAAANGATGG		ENKRRYQ (D)
4		NGGNGTTTCNACACCNTGNAA	FQGVETP (D)
5	TATGTCGTTCCCATCTGCAG		MSFPSA (Cp)
6	AAACAATCCTTCTACAGGAA		KQSFYR (Cp)
7	AAGCAGATATGTTGGGTACT		SRYVGY (Cp)
8	TAAGTACAAGTTGCAACTCC		KYKLQL (Cp)
9	GTTTCCTTTGCGCTCTCAAA		FESAKE (Cp)
10		AGTTGACCATTCTCGGTATA	IPRMVN (Cp)
11		ACCCTTCTACAAAGATACGC	RIFVEG (Cp)
12	GATGACCTGGCGTAGCAGC		5′UTR (Cp)
13		AAATCGGAAAGCTTCTTGATG	3′UTR (Cp)
14	ATGCCAGTGGTGAGATACGA		MPVVRY (Cp)
15		TCAAGGCTCAATGAATATCACCTCCACT	AVEVIFIEP (Cp)
16	AGATGGGAATGACATAGCAATAACACGATC		DRVIAMSFPS (Hn)
17	CCTCAGACGAGTTGACCTAGACACATCAGC		5′UTR (Hn)
18		CTGCTATGAGTGAGTAACACCTCGTTTAGC	3′UTR (Hn)
19	ATGTCATCAGTAAGATATGACTCTGGGTCG		MSSVRYDSGS (Hn)
20		TTAGCATACATTAATTACCTTGCA	CKVINVC stop (Hn)
21	CAAATATTCCACAGATGGTGATCTTTCTGC	GCAGAAAGATCACCATCTGTGGAATATTTG	NIPQMVIFL (Cp‐VSP R153Q)
22	ACAAATATACCGCAGATGTGCAACTTCCTG	CAGGAAGTTGACCATCTGCGGTATATTTGT	TNIPQMCNFL (Hn‐VSP R153Q)
23	GCAATTCACTCGAAAGGCGGA	TCCGCCTTTCGAGTGAATTGC	AIHSKGG (Cp‐VSP C302S)
24	CACTACAGGGTGGAGCGGATC		HYRVERI (Cp)
25		ATCGCACTTTCACATCGCCAC	GDVKVR (Cp)
26	CCCTGTATGCCTCTGGTCGTACAAC		LYASGRT *β* actin (Cp)
27		CAAGATCTTCATCAGATAGTCGGTC	TDYLMKIL *β* actin (Cp)
28	GCGTGACTGGATGGCACAGGATCAGA		RDWMAQDQ (Hn)
29		CCCATTGTTTTTCCGACACCTTGAATTGAATT	NSIQGVGKTM (Hn)
30	AAGATCTGGCANCACACNTTCTACAA		KIWXHXFY *β* actin (Hn)
31		GATCCACATCTGNTGGAAGGTGGA	STFXQMWI *β* actin (Hn)

Underlined codons indicate the mutated sites. Cp, *Cynops pyrrhogaster*; Hn, *Hynobius nebulosus*; D, degenerate primers.

For cloning of salamander VSP cDNA, total RNA was isolated from the testis of an adult salamander (*H. nebulosus*) collected from a pond in the vicinity of Yamaguchi city, Japan. Isolation of partial cDNA fragment using degenerative PCR primers was performed similarly as for newt VSP. A RACE protocol was used to amplify the 5′ end by synthesizing a first strand using gene‐specific primer #16. An ORF of 1284 bp was amplified with primer #17 and 18 followed by nested PCR with primers #19 and 20.

### Amino acid sequence and phylogenetic analysis

The cDNA sequences and their deduced amino acids of Cp‐VSP and Hn‐VSP were analyzed using Genetyx‐Win Version 4.0. Pairwise alignment with Ci‐VSP was performed with Emboss stretcher (http://www.ebi.ac.uk/Tools/psa/emboss_stretcher/). Multiple amino acid alignment was performed with ClustalW2 (www.ebi.ac.uk/Tools/msa/clustalw2/). The dendrogram tree was prepared by using the neighbor‐joining using ClustalX and drawn by Njplot. Genbank accession numbers of VSP orthologs were 148230800 (*Xenopus laevis*) and 118084924 (*Gallus gallus*).

### Molecular constructs and site‐directed mutagenesis

Salamander and newt VSPs genes and their mutants were subcloned with *NotI* and *XhoI* into the *Xenopus* oocyte expression vector pSD64TF (kindly gifted by Dr. Terry Snutch). All mutants (Cp‐VSP R153Q, Cp‐VSP C302S, Hn‐VSP R153Q, C2‐domain truncated Ci‐VSP, and Cp‐VSP) were made by using the QuikChange kit (Stratagene, La Jolla, CA) and confirmed by sequencing of the ORFs. A mMESSAGE mMACHINE transcription kit (Ambion, Carlsbad, CA) was used to synthesize cRNAs, which were stored at −80°C until use. Cp‐VSP constructs in pSD64TF were linearized with *EcoRI*, while those of Hn‐VSP were linearized with *XbaI*.

### Analysis of gene expression pattern of Cp‐VSP and Hn‐VSP by RT‐PCR

A female newt was anesthetized for 15 min with a 0.2% ice‐cold aqueous solution of ethyl 3‐aminobenzoate methanesulfonate salt (Sigma‐Aldrich, St. Louis, MO). Brain, heart, lung, stomach, kidney, liver, ovary, muscle, eye, retina, and spleen tissue samples were collected and each was divided into two portions. For retinal pigment epithelium, a newt was anesthetized and the eyes are dissected out. By peeling away the sclera from the enucleated eyes with fine forceps, a small sheet of RPE was obtained. Total RNA from each sample was isolated with TRIzol^®^ reagent according to the manufacturer's procedure. The integrity of the RNA was determined by measuring the optical absorbance of 260 nm and 1% gel electrophoresis. One microgram of the total RNA was reverse transcribed with oligo(dT)_20_. For semiquantitative RT‐PCR, a 600‐bp fragment of Cp‐VSP was amplified from the cDNA pool with primers #24 and 25 and a 150‐bp fragment of *β*‐actin was amplified with primers #26 and 27.

A male salamander was anesthetized as described above and testis, brain, heart, lung, stomach, kidney, spleen, liver, muscle, and eye tissues were collected. A 300‐bp fragment of Hn‐VSP was amplified from the cDNA pool with primers #28 and 29 and a 650‐bp fragment of *β*‐actin was amplified with degenerate primers #30 and 31.

### Electrophysiological recordings

All experiments were performed according to the guidelines of the Animal Research Committees of Graduate School of Medicine of Osaka University. *Xenopus laevis* was anesthetized as described above and ovary tissue was collected surgically and treated with 1 mg/mL collagenase (Sigma‐Aldrich) for up to 2 h at room temperature. Defolliculated oocytes were injected with about 50 nL cRNA per oocyte and incubated for 2–4 days at 18 °C in ND96 solution (5 mmol/L HEPES, 96 mmol/L NaCl, 2 mmol/L KCl, 1.8 mmol/L CaCl_2_, 1 mmol/L MgCl_2_, pH 7.5) supplemented with pyruvate and gentamycin (Goldin 1992).

Under two‐electrode voltage clamp (TEVC), “sensing” currents were measured as previously described (Murata et al. 2005; Sakata et al. 2011) with a “bath‐clamp” amplifier (OC‐725‐HV; Warner Instruments, Hamden, CT). Stimulation and data analysis were done on a Mac mini computer using an LIH 8+8 AD/DA converter and Patchmaster software (HEKA Electronik, Lambrecht, Germany). Intracellular microelectrodes of resistance up to 0.6 MΩ were fabricated from borosilicate glass capillaries (GC150TF‐10, Harvard Apparatus Ltd, Kent, UK) using a P‐97 horizontal puller (Sutter Instruments Co., Novato, CA) and filled with 3 mol/L KCl, pH 7.2. Bath solution was composed of 96 mmol/L *N*‐methyl‐d‐glucamine (NMDG) – methanesulfonate, 5 mmol/L HEPES, and 3 mmol/L MgCl_2_, pH 7.2. A holding potential was set at −60 mV and steps were applied with each 10 mV increment for 21 pulses (Figs. 2A, 3A). Linear current components including leak currents and current as a result of charging cell capacitance were subtracted with a P/‐5 protocol and the moved charge (Q_off_) was obtained by the integration of the Off “sensing” current. Upon voltage depolarization at more positive than 100 mV, endogenous outward currents were superimposed on the On “sensing” currents. Off “sensing” currents were similar to those recorded with the cut‐open oocyte system (data not shown). The charge–voltage relationship (Q‐V) curves were fitted by a Boltzmann equation: Q = 1/(1 + exp (−*z*F(V−V_1/2_)/RT)), where *z* is the effective valence and V_1/2_ is the voltage at which half of the charge moves across the plasma membrane. To monitor phosphatase activity of VSPs using GIRK2 currents as the read out, cRNAs of VSP were coinjected with those of GIRK2d, a version of GIRK2 (kindly provided by Dr. Kurachi), G*β*1 and *γ*1 in the ratio 1:1:1:1. GIRK2 currents were measured in bath solution containing 25 mmol/L NMDG, 3 mmol/L MgCl_2_, 10 mmol/L HEPES, 80 mmol/L KOH, pH 7.3. GIRK2 currents were measured using step pulses to −100 mV, with interval voltage steps to 50, 65, and 90 mV to activate VSP activity (Fig. 4B). The oocytes were allowed to rest for 5 min between series to allow the phosphoinositide pool to recover before the next series. All of the data were analyzed by Igor Pro 6.0 (Wavemetrix Inc. Portland, OR) software and the experiments were done at 22–25°C.

### Statistical analysis

Data are given as means ± SD or ± SEM where applicable. *N* indicates the number of oocytes for particular set of data. Test for significance was performed with unpaired Student's *t*‐test. The V_1/2_ values were calculated using the Boltzmann equation to fit the curves of the maximum current amplitudes plotted over the test potentials to activate these currents. Mean V_1/2_ values from WT VSPs were compared with those of R153Q mutants and tested for statistical significance at *P* value <0.0001. Mean values for the GIRK2 current decay at 3 sec for the different test potentials were compared and then tested for statistical significance at *P* value <0.05.

## Results

### Amino acid sequences of newt VSP (Cp‐VSP) and salamander VSP (Hn‐VSP)

**Figure 1.**
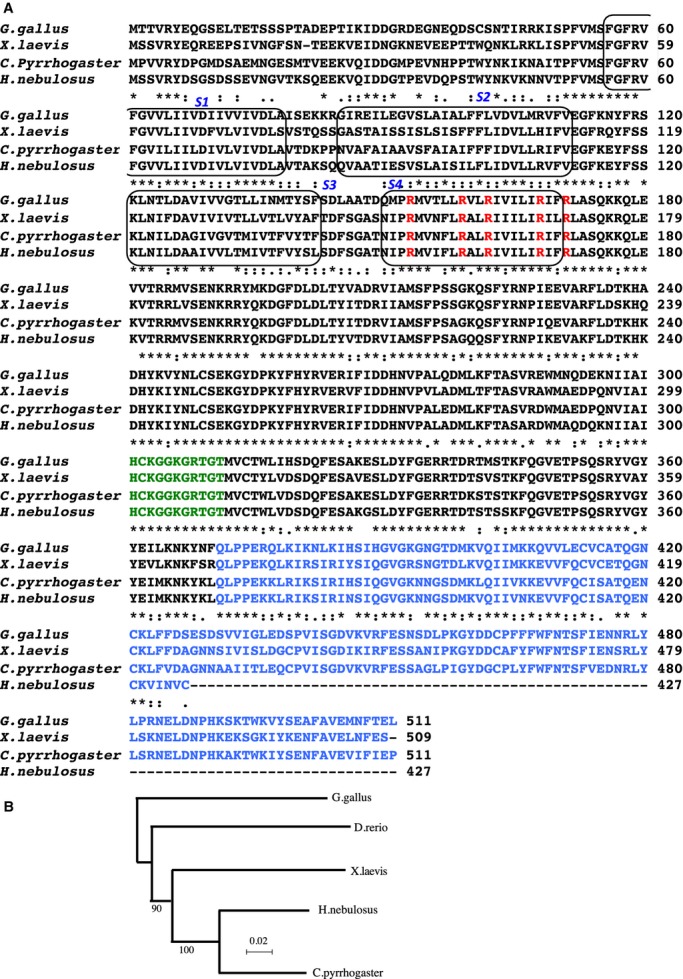
(A) Multiple amino acid alignments of newt (*Cynops pyrrhogaster*), salamander (*Hynobius nebulosus*), frog (*Xenopus laevis*), and chick (*Gallus gallus*) VSPs. The transmembrane segments (S1–S4) are surrounded by round squares. The conserved arginines in the S4 segment are shown in red. The conserved active site of the phosphatase domain is shown in green. The C2 domain is shown in blue. Note that a part of C2 domain is absent in salamander VSP. (B) Dendrogram of amino acid sequences of VSP orthologs. Bootstrap value is indicated.

The full‐length Cp‐VSP cDNA sequence was 2264 bp. The ORF was 1536 bp, encoding a protein of 511 amino acids. Hn‐VSP cDNA sequence was 1708 bp which comprised 1284 bp ORF encoding a protein of 427 amino acids. Multiple sequence alignment with other known VSPs from *X. laevis* and *G. gallus* demonstrated conservation of the multiple, positively charged arginines in the S4 segment that are intervened by two hydrophobic residues (Fig. 1A), which is a signature motif conserved in all VGICs and VSPs (Okamura et al. 2009). In addition, the phosphatase domains harbor the active site containing cysteine (HCKGGKGRTGT; shown in green in Fig. 1A), similar to that found in PTEN. This cysteine in the active site provides a thiophosphate bond during substrate catalysis in all protein–tyrosine phosphatases and phosphoinositide phosphatases (Maehama and Dixon 1998; Maehama et al. 2001). The protein sequences of Hn‐VSP and Cp‐VSP showed 70.0% amino acid identity with Gg‐VSP.

We noted that the C2 domain (shown in blue in Fig. 1A) of Hn‐VSP is truncated after the 427th amino acid. This was not due to an artifact in the process of cloning, since the poly (A)+ tail was present in the mRNA and multiple independent cDNA clones from testis, spleen, and kidney showed the same truncation. Phylogenetic analysis demonstrated that newt and salamander VSPs are evolutionarily more closely related to each other than to *X. laevis* VSP and *G. gallus* VSP (Fig. 1B).

### Cp‐VSP and Hn‐VSP show voltage‐driven charge movement across the plasma membrane

**Figure 2.**
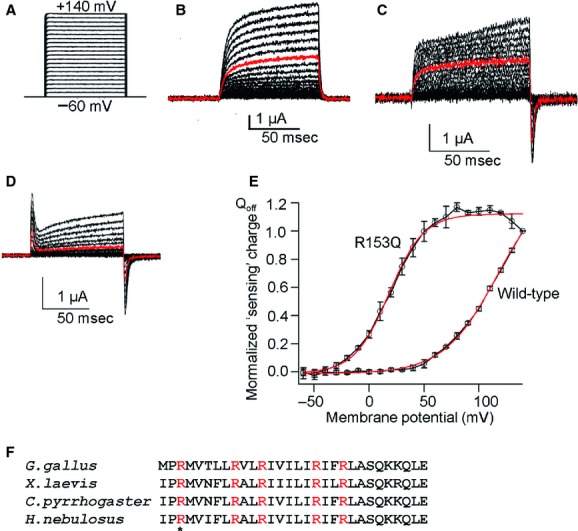
Measurement of Off “sensing” currents in newt VSP (Cp‐VSP) and its R153Q mutant. (A) A pulse protocol. Voltage was evoked ranging from −60 to +140 by 10‐mV increments. (B) Representative traces of an uninjected oocyte. (C, D) Representative traces of Off “sensing” currents of wild‐type (WT) Cp‐VSP and its R153Q mutant, respectively. Red trace indicates the current at +80 mV. Both On “sensing” and Off “sensing” currents were observed in R153Q mutants. (E) Q_off_‐V curves from WT Cp‐VSP and its R153Q mutant. Red curves indicate fit by a Boltzmann equation. In WT data, plots were not saturated. The value of charges at 140 mV was used for standardization of Q_off_‐V curves for WT. V_1/2_ of the R153Q mutant was 18.1 ± 1.5 mV, *n* = 4. Error bars are mean ± SD. Charge was standardized by the charge value of Q_off_ at repolarization from +140 mV step pulse. (F) Alignment of amino acid sequences of S4 segment among Cp‐VSP, Hn‐VSP, Xl‐VSP, and Gg‐VSP and the position of R153 is shown by asterisk.

**Figure 3.**
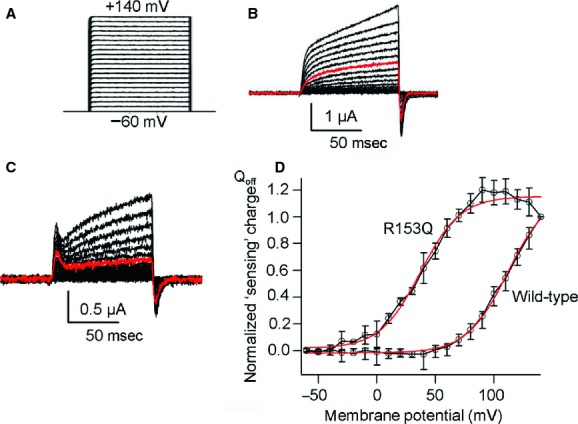
Off “sensing” currents in salamander VSP (Hn‐VSP) and its R153Q mutant. (A) A pulse protocol. Voltage was evoked ranging from −60 to +140 by 10‐mV increments. (B, C) Representative traces of Off “sensing” currents of wild‐type (WT) Hn‐VSP and its R153Q mutant, respectively. Red trace indicates the current at +80 mV. Both On “sensing” and Off “sensing” currents were observed in the R153Q mutant. (D) Q_off_‐V curves from WT Hn‐VSP and its R153Q mutant. Red curves indicate plots fitted by Boltzmann equation. V_1/2_ of the R153Q mutant was 37.0 ± 2.6 mV, *n* = 4.

To examine whether both Cp‐VSP and Hn‐VSP have functional VSD as known in other VSP orthologs, including Ci‐VSP from ascidian (Murata et al. 2005), Dr‐VSP from zebrafish (Hossain et al. 2008), Gg‐VSP from chick (Kurokawa et al. 2012), and Xl‐VSP from African clawed frog (Ratzan et al. 2011), we examined their “sensing” currents which are expected to be elicited as the movement of charges associated with the voltage‐dependent conformational change in the VSD. Uninjected *Xenopus* oocytes showed neither On nor Off “sensing” currents (Fig. 2B). Oocytes microinjected with cRNA of Cp‐VSP or Hn‐VSP showed robust Off “sensing” currents (Figs. 2C, 3B). Charges calculated from Off “sensing” currents were plotted against voltage (Q_off_‐V plot). The Q_off_‐V plots from wild‐type (WT) and their R153Q mutants were fitted with Boltzmann equation (Fig. 2E for Cp‐VSP and Fig. 3D for Hn‐VSP). Both WT Cp‐VSP and Hn‐VSP showed unsaturated charge movement.

It is known in VGICs and VSPs that positively charged residues on the S4 segment play a critical role in voltage sensing. Mutation of arginine to glutamine at the residue in other VSPs which corresponds to arginine 153 of Hn‐VSP and Cp‐VSP (Fig. 2F) has been shown to negatively shift the voltage‐dependent motion of the VSD (Hossain et al. 2008; Kohout et al. 2008; Ratzan et al. 2011). We therefore mutated the arginine (R) in position 153 of both Cp‐VSP and Hn‐VSP to glutamine (Q) and measured their Off “sensing” currents (Figs. 2D, 3C). In most cases, On “sensing” currents were also observed. Q_off_‐V plots from these mutants showed that voltage dependence was negatively shifted (Figs. 2E, 3D). Both showed faster kinetics of “sensing” currents compared to their WTs. In addition, in Q_off_‐V curves of the R153Q mutants the amount of charges was saturated at higher membrane potentials, which confirm that the observed currents were in fact derived from the motions of the VSD both in Cp‐VSP and Hn‐VSP.

### Hn‐VSP does not exhibit voltage‐dependent phosphatase activity

**Figure 4.**
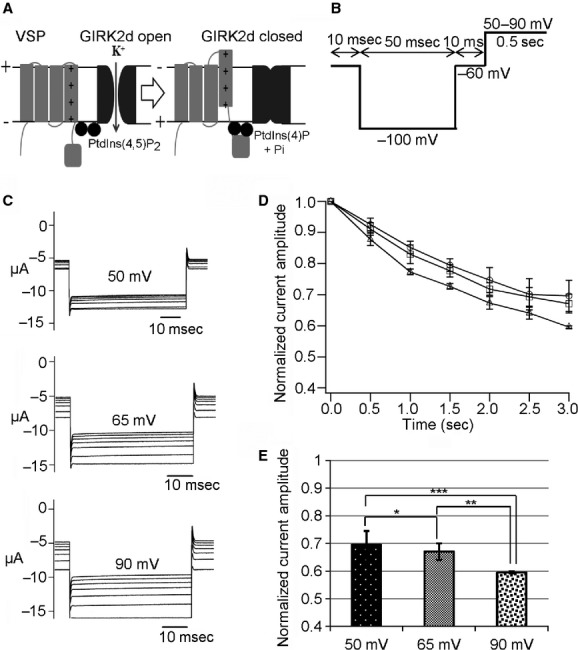
Measurements of phosphatase activity of newt VSP (Cp‐VSP). (A) Schematic representation of experimental GIRK2 read out of depolarization‐dependent VSP activity. PtdIns(4,5)P_2_ depletion leads to the closure of the GIRK2 ion channel. (B) A pulse protocol for measuring GIRK2 currents on newt and salamander VSPs. Interval voltages were set to 50, 65, and 90 mV. (C) Depolarization‐dependent phosphatase activity of Cp‐VSP recorded at 50, 65, and 90 mV. (D) The amplitudes of the inward GIRK2 currents were normalized to the value of the first current trace and plotted against time. Data in the plot are mean ± SEM, *n* = 6, 50 mV (circles), 65 mV (squares), and 90 mV (triangles). (E) Bar graph for the normalized current at 3 sec. Error bars are SEM, *P = 0.68, **P = 0.04, ***P = 0.02.

Depolarization‐dependent phosphatase activities of both Cp‐VSP and Hn‐VSP were investigated by monitoring conductance of GIRK2 inward rectifier potassium channel using TEVC in the *Xenopus* oocyte. The GIRK2 channel is known to require PtdIns(4,5)P_2_ for its activation, and PtdIns(4,5)P_2_ is depleted by the enzymatic activities of VSPs (Fig. 4A; Murata et al. 2005; Hossain et al. 2008; Ratzan et al. 2011). When coexpressed with Cp‐VSP, GIRK2 currents decreased remarkably as the interval voltage was increased from 50 to 90 mV, as shown in Figure 4C. Normalized amplitudes of GIRK2 current decay due to Cp‐VSP activities were plotted against time (Fig. 4D). Such a depolarization‐dependent decrease in GIRK2 currents was not observed when coexpressed with the enzyme‐inactive C302S mutant of Cp‐VSP (data not shown).

**Figure 5.**
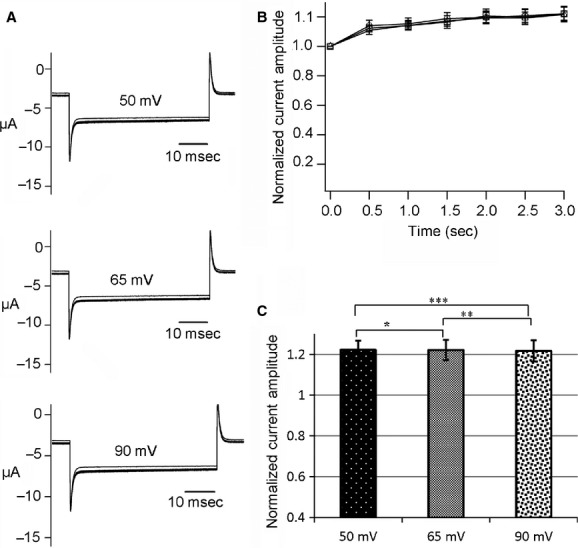
Measurements of phosphatase activity of salamander VSP (Hn‐VSP). (A) Phosphatase activity of Hn‐VSP recorded at 50, 65, and 90 mV. (B) Normalized current amplitudes plotted against time. Data in the plot are mean ± SEM, *n* = 4, 50 mV (circles), 65 mV (squares), and 90 mV (triangles). (C) Bar graphs for current amplitudes at 3 sec. Error bars are SEM, *P = 0.99, **P = 0.94, ***P = 0.95.

On the other hand, recordings from the oocytes coinjected with Hn‐VSP and GIRK2 cRNAs displayed no observable decrease in GIRK2 currents (Fig. 5). Amplitudes of inward GIRK2 currents at 3 sec from the initiation of the pulse protocol are demonstrated as bar charts for both Cp‐VSP (Fig. 4E) and Hn‐VSP (Fig. 5C). These were significantly different among the three depolarizing potentials in Cp‐VSP while those from Hn‐VSP were not (Figs. 4E, 5C).

### The C2 domain is essential for phosphatase activity of VSP

**Figure 6.**
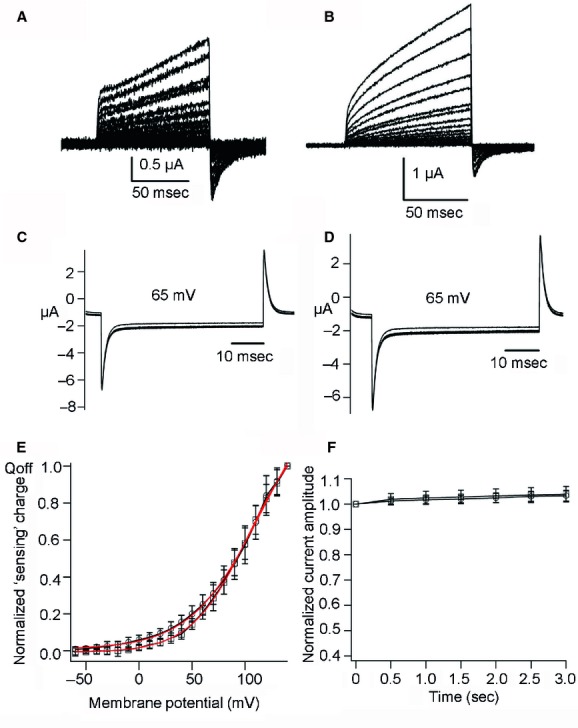
C2‐domain truncated Ci‐VSP and Cp‐VSP mutants lack phosphatase activity. (A, B) Sensing currents recording of C2‐domain truncation mutant of Ci‐VSP and Cp‐VSP, respectively. (C, D) Phosphatase activity of truncated mutant of Ci‐VSP and Cp‐VSP, respectively, measured at 65 mV. (E) Q_off_‐V plots for truncated Ci‐VSP (squares) and Cp‐VSP (circles), *n* = 6 for both. (F) Normalized current amplitudes plotted against time, *n* = 4 for both Ci‐VSP (squares) and Cp‐VSP (circles). Data in the plots are mean ± SD.

To probe further whether the loss of phosphatase activity in Hn‐VSP was due to truncation in the C2 domain, a similar truncation corresponding to the same position of Hn‐VSP was introduced into Ci‐VSP and Cp‐VSP. In TEVC recording (Fig. 6A and B) from either oocyte microinjected with cRNA encoding truncated Ci‐VSP or truncated Cp‐VSP, the off “sensing” charge was detected, verifying that the C2‐domain truncated versions of Ci‐VSP and Cp‐VSP were expressed on the cell surface. These truncated versions of VSPs did not show voltage‐dependent decrease in GIRK2 channel activities in *Xenopus* oocytes (Fig. 6C–F), suggesting that they do not have phosphatase activity.

### Tissue mRNA expression profile of the Cp‐VSP and Hn‐VSP genes

**Figure 7.**
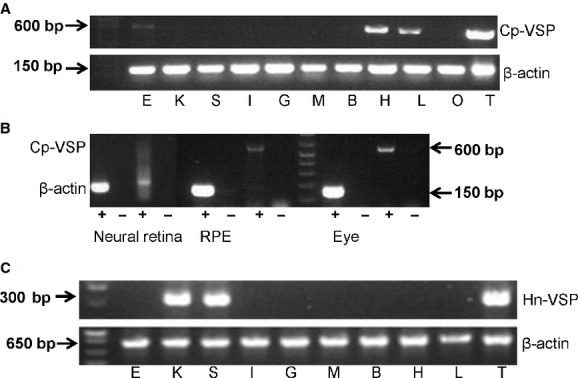
RT‐PCR results of the gene expression pattern of Cp‐VSP and Hn‐VSP. (A) Cp‐VSP (upper panel). *β*‐actin for the positive control (lower panel). E, eye; K, kidney; S, spleen; I, intestine; G, gut; M, muscle; B, brain; H, heart; L, lung; O, ovary; T, testis. (B) Expression of Cp‐VSP in eye and retinal pigment epithelium (RPE), but not in the neural retina (+ or − indicates PCR reaction with or without reverse transcriptase) (C) Hn‐VSP (upper panel). *β*‐actin for the positive control (lower panel). E, eye; K, kidney; S, spleen; I, intestine; G, gut; M, muscle; B, brain; H, heart; L, lung; O, ovary; T, testis. Arrows indicate band size.

RT‐PCR was performed to investigate the tissue expression pattern of both Cp‐VSP and Hn‐VSPs (Table 2). Results indicated that Cp‐VSP was shown to be expressed in testis, heart, lung, and eye (Fig. 7A). Further analysis of the eye in newt revealed that Cp‐VSP was expressed in the retinal pigment epithelium (RPE), but not in the neural retina (Fig. 7B). Hn‐VSP was found to be expressed in testis, kidney, and spleen (Fig. 7C). We failed to detect gene expression in ovary of Cp‐VSP unlike in *Xenopus* (Ratzan et al. 2011). This was consistent with our previous finding that there is no endogenous activity of VSP in *Cynops* oocyte, whereas *Xenopus* oocyte has endogenous VSP activities (Kurokawa et al. 2012; Liu et al. 2012). We did not test gene expression of ovary in *Hynobius nebulosus*.

**Table 2.** Summary of expression pattern of Cp‐VSP and Hn‐VSP

Tissue	Newt (Cp)	*n*	Salamander (Hn)	*n*
Testis	+	4	+	6
Ovary	−	3	NT	
Heart	+	4	+	2
Lung	+	4	−	1
Kidney	−	5	+	4
Stomach	−	4	−	1
Muscle	−	6	−	2
Liver	−	6	−	2
Brain	−	3	−	2
Eye	+	6	−	2
RPE	+	1	NT	
Neural retina	−	1	NT	
Spleen	−	3	+	5

NT, not tested; +, VSP present; −, VSP absent. VSP, voltage‐sensing phosphatase; RPE, retinal pigment epithelium.

## Discussion

In this study, VSP orthologs were cloned from two species of urodele amphibians. Bioinformatic searches of the ORFs confirmed that they belonged to the VSP family, and the overall structure was similar to those previously reported, except for the truncation in the C2 domain of Hn‐VSP. RT‐PCR cloning from other tissues resulted in isolation of the same cDNA sequence, making it unlikely that truncation in the C2 domain was due to biased cDNA cloning of minor population of alternatively spliced variants. Further detailed information of genome sequence will be necessary to confirm whether DNA sequence corresponding to the C2 domain is absent in Hn‐VSP gene.

Wild‐type Hn‐VSP and Cp‐VSP exhibited only Off “sensing” currents as opposed to On and Off “sensing” currents of their R153Q mutants (Figs. 2C and D, 3B and C). The shift of Q_off_‐V curve to a less positive potential was found in the R153Q mutants compared with the WTs, which is in agreement with the similar mutant of previously reported VSP orthologs (Hossain et al. 2008; Kohout et al. 2008; Ratzan et al. 2011). We interpret that the apparent absence of On “sensing” currents in WTs was due to broad timing of charge motion during depolarization. These findings indicate that VSDs of both VSPs are functional.

Both Cp‐VSP and Hn‐VSP showed that the motions of their VSDs further increased at membrane potentials over 100 mV. It is unlikely that such high depolarization is attained in physiological conditions. However, we cannot rule out the possibility of posttranslational modifications or additional cofactors which could make the VSPs operate in a more physiological voltage range. For example, it has been reported that human and mouse voltage‐gated proton channels (Hv1/VSOP), when heterologously expressed, were observed to open at more negative potentials than those endogenously expressed in native cells (Musset et al. 2008). It is possible that entire range of voltage sensitivity of VSPs is not reached in physiological context and full phosphatase activity is not utilized to exert its physiological action.

We found that Cp‐VSP showed voltage‐dependent phosphatase activity. At a membrane potential of +50 mV, currents through GIRK2 channels coexpressed with Cp‐VSP were mildly reduced, whereas these were more remarkably reduced at +90 mV, reaffirming the notion that capacity of catalyzing phosphoinositides by Cp‐VSP is increased during depolarization as known in other VSPs (Murata and Okamura 2007).

On the other hand, Hn‐VSP lacking the C2 domain did not exhibit phosphatase activity. Recent crystal structures of Ci‐VSP showed the overall similarity of structure between PTEN and the cytoplasmic region of Ci‐VSP (Matsuda et al. 2011; Liu et al. 2012). The overall structure of the C2 domain of Ci‐VSP does not remarkably differ from that of PTEN except for a difference at the region called “CBR3 loop” that plays a role in stabilizing the protein through association with the membrane in PTEN (Lee et al. 1999). Frequently occurring mutations in the C2 domain of human PTEN have been reported in cancer patients (Maehama et al. 2001). Mutating basic amino acids or truncation in the C2 domain of PTEN in vitro leads to loss of tumor suppressing activities and the phosphatase activities (Georgescu et al. 1999, 2000; Lee et al. 1999). The C2 domain has been suggested to play role in orienting the phosphatase domain into contact with the plasma membrane for dephosphorylating PtdIns(3,4,5)P_3_ (Lee et al. 1999; Georgescu et al. 2000). From analogy to PTEN, proper orientation of the phosphatase domain to allow binding substrate may also require the C2 domain in VSP and this may not take place in the versions of VSPs lacking part of the C2 domain. The C2 domain is also known to be important for protein stability: truncation or mutation in the C2 domain in PTEN leads to higher proportion of degradation of the protein (Georgescu et al. 1999, 2000). This probably does not apply to VSP, since sensing currents were observed both from Hn‐VSP and C2‐domain truncated versions of Cp‐VSP and Ci‐VSP. Taken together, the C2 domain plays a crucial role in phosphatase activity of VSP and the absence of phosphatase activity of Hn‐VSP is due to the truncation in the C2 domain.

Functional significance of a noncatalytic VSP encoded in the *H. nebulosus* genome is unclear. It has been reported that some of the myotubularin family members of phosphoinositide 3‐phosphatases do not have their phosphatase activity but play regulatory roles (Robinson and Dixon 2006). It is possible that C2‐domain truncated VSP of salamander may exert voltage‐dependent activity through regulating potential binding partner at the truncated cytoplasmic region or the N‐terminal region. It has recently been shown that the N‐terminus of Ci‐VSP moves upon membrane depolarization (Tsutsui et al. 2013).

VSP cDNAs were identified from testes of two urodeles. Urodeles display diverse mechanisms for the prevention of polyspermy. In *H. nebulosus*, which exhibits monospermic fertilization, the fast block to polyspermy is regulated by a positive fertilization potential of the egg plasma membrane, whereas *C. pyrrhogaster* exhibits physiological polyspermy and electrical regulation is absent or limited only to dejellied eggs (Appendix; Iwao and Jaffe 1989; Iwao et al. 1994). It is thought that in such a block, a second sperm is able to sense the depolarized fertilization potential of the fertilized egg and be prevented from fusing with the egg membrane (Iwao and Jaffe 1989; Appendix). Previous characterization of *Xenopus* VSPs showed that the voltage‐dependent activation of phosphoinositide phosphatase matched well with the reported voltage dependence of polyspermy block in frog (Ratzan et al. 2011), raising a possibility that VSPs might establish the basis for the fast block to polyspermy. However, noncatalytic VSP in salamander is not consistent with this hypothesis.

VSP gene was found to be expressed in the RPE of the newt eye. Newts have the ability of regenerating some of their damaged or removed tissues as known in examples including regeneration of the retina through transdifferentiation of the RPE cells (Cheon et al. 1998), lens (Reyer 1954), heart (Oberpriller and Oberpriller 1974) as well as limbs and tail (Niazi et al. 1985). RPE plays an important role in the visual system and express numerous ion channels. Defects in these ion channels have been shown to affect RPE cell physiology and lead to degenerative disease of the retina (Wimmers et al. 2007). In addition, bioelectrical signaling has been shown to control tissue shape and structure during the process of regeneration and tumorigenesis (Levin 2013). Further studies are required for understanding biological roles of VSPs.

## Acknowledgments

The authors thank Drs Yoshimitsu Miyoshi and Hajime Sawai for their help in preparation of RPE of newt eyes, Dr. Yoshihisa Kurachi, Medical School, Osaka University for kindly providing GIRK2d clone, Dr. L. A. Jaffe, Department of Cell Biology, University of Connecticut Health Center, CT, USA, and Dr. William Ratzan for discussion, insightful comments and critical reading of the manuscript.

## Conflict of Interest

None declared.

## Accession Numbers

Accession numbers of cDNA sequences of newt VSP (Cp‐VSP) and salamander VSP (Hn‐VSP) are AB889939 and AB889940, respectively.

## Figures and Tables

**Figure 8.**
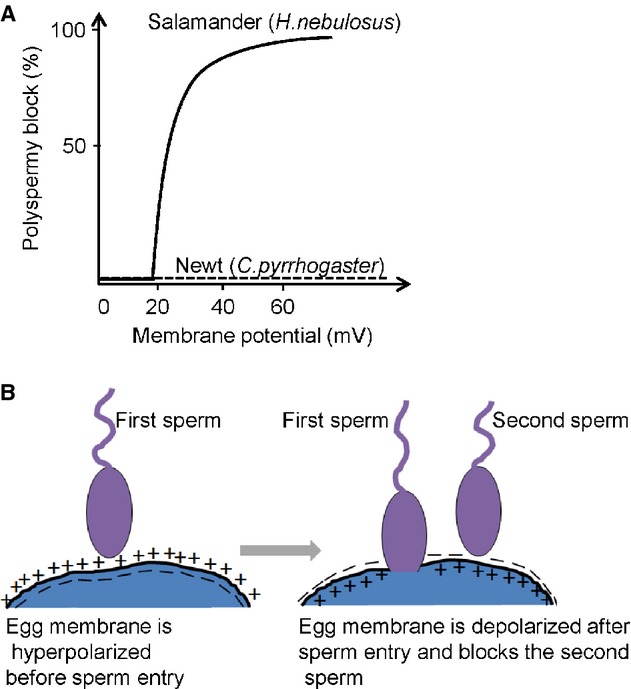
Positive‐going membrane potential controls sperm‐egg fusion in *Hynobius nebulosus* but not in *Cynops pyrrhogaster*. (A) A graphical representation of percent polyspermy block. When the eggs were clamped at membrane potentials >20 mV Hn‐sperms (curve) were prevented from fusing with the egg, whereas Cp‐sperms (dash line) were not (from Iwao and Jaffe 1989). (B) Cartoon showing the hypothesis of molecular mechanisms of polyspermy block, based on the ability of sperm to sense change in the egg membrane potential (adapted from Iwao and Jaffe 1989). After the first sperm is successfully fused with egg, the second sperm is prevented from fusion with the egg through VSP activities. In the second sperm, VSP, which could potentially be localized in sperm membrane, senses depolarized egg cell membrane through transient cell fusion and its phosphatase activity downregulates phosphoinositides which may be required for progression of cell fusion event (Ratzan et al. 2011).
